# Evolution, function and roles in drug sensitivity of trypanosome aquaglyceroporins

**DOI:** 10.1017/S0031182021000354

**Published:** 2021-09

**Authors:** Juan F. Quintana, Mark C. Field

**Affiliations:** 1 Wellcome Centre for Integrative Parasitology (WCIP), Institute of Biodiversity, Animal Health and Comparative Medicine (IBAHCM), University of Glasgow, Glasgow G61 1QH, UK; 2 School of Life Sciences, University of Dundee, Dundee DD1 5EH, UK; 3 Institute of Parasitology, Biology Centre, Czech Academy of Sciences, 37005 Ceske Budejovice, Czech Republic

**Keywords:** Aquaglyceroporin, drug resistance, membrane trafficking, pentamidine, sleeping sickness, *Trypanosoma brucei*

## Abstract

Aquaglyceroporins (AQPs) are membrane proteins that function in osmoregulation and the uptake of low molecular weight solutes, in particular glycerol and urea. The AQP family is highly conserved, with two major subfamilies having arisen very early in prokaryote evolution and retained by eukaryotes. A complex evolutionary history indicates multiple lineage-specific expansions, losses and not uncommonly a complete loss. Consequently, the AQP family is highly evolvable and has been associated with significant events in life on Earth. In the African trypanosomes, a role for the AQP2 paralogue, in sensitivity to two chemotherapeutic agents, pentamidine and melarsoprol, is well established, *albeit* with the mechanisms for cell entry and resistance unclear until very recently. Here, we discuss AQP evolution, structure and mechanisms by which AQPs impact drug sensitivity, suggesting that AQP2 stability is highly sensitive to mutation while serving as the major uptake pathway for pentamidine.

## Graphical abstract

**Figure:**
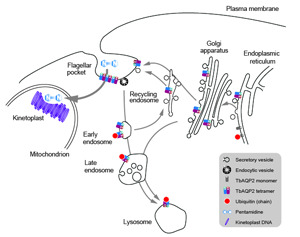

## Introduction

**Fig. 1.**
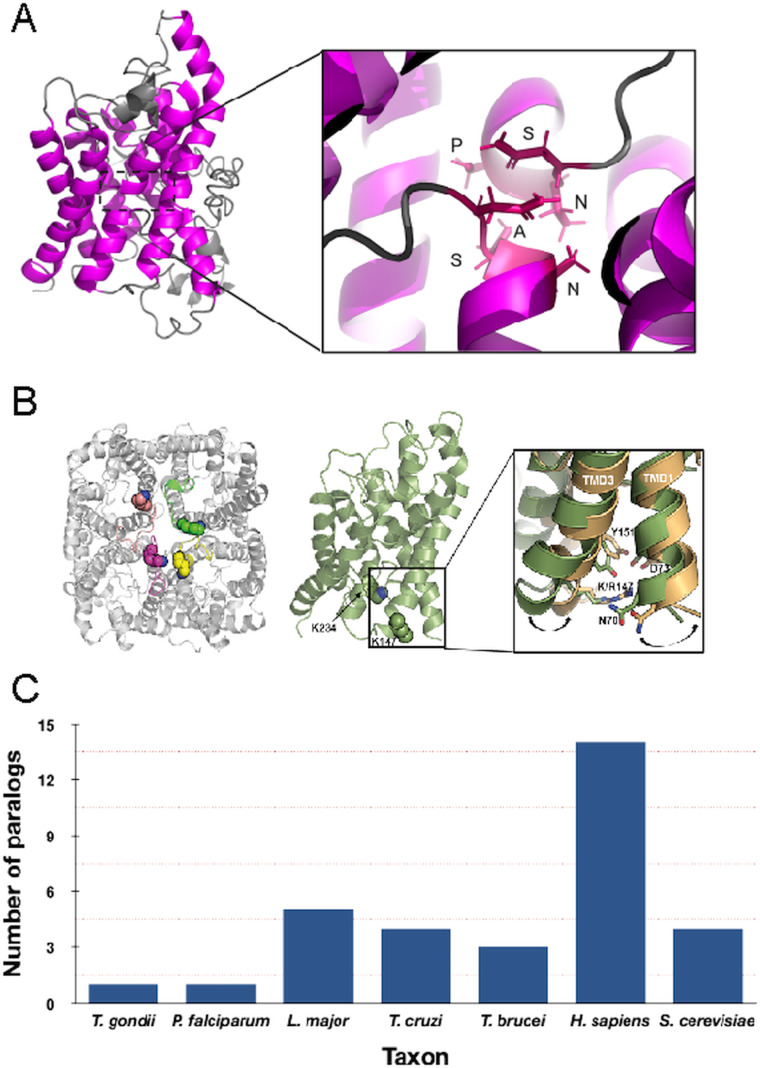
Structure and copy number of AQP paralogues. (A) Left panel: Depiction of the *Trypanosoma brucei* AQP2 monomer. The *trans*-membrane domains are highlighted in magenta. Right panel: Details of the unique NPS/NSA TbAQP2 selectivity pore. (B) Left panel: Top view of the proposed tetrameric structure of *T*. *brucei* AQP2 model. The lysine residues in position K147 and K234 are shown as spheres. Right panel: Expanded view of the conformational change observed during TMD simulations on TMD1 and TMD3 as a result of the K147R mutation. Wild type TbAQP2 is shown in green. TbAQP2 displaying the K147R and K234R mutations is shown in light orange. Other residues important for intramolecular interactions between transmembrane domains (N70, D73, K142 and Y151) are also highlighted. Mutations on these residues profoundly impair protein stability, rendering the parasites resistant to pentamidine and melarsoprol. (Quintana *et al*., 2020). (C) Number of clear AQP paralogues detected in representative taxa. Note that for the protists these are all represented by the more permissive glycerol-capable class.

Aquaglyceroporins (AQPs) were first identified in the 1990s as membrane proteins with functions in osmoregulation and the translocation of low molecular weight solutes, including glycerol and urea (Preston *et al*., 1992). In humans, dysfunction is associated with multiple cancers, kidney disease, oedema and other pathologies (King *et al*., 2004; Yool *et al*., 2010; Shi *et al*., 2012). AQPs have an evolutionarily broad representation, being found in most pro- and eukaryotic taxa and they retain a conserved architecture encompassing six hydrophobic domains. This structure is in turn derived through an internal duplication from a primordial protein with three membrane-spanning helices, reflected in the presence of two NPA (Asn-Pro-Ala) boxes that are involved in channel functions. Both the N- and C-termini face the cytoplasm (Fig. 1) and sequence and architectural conservation indicates vertical descent. Consequently, at least one mechanism for the control of water (and solute) passage across biological membranes arose very early in the history of life (Ishibashi *et al*., 2020). However, AQPs are not present in all taxa, for example the bacterial phyla *Fibrobacteres* and *Lentisphaerae*, as well as some parasites and extremophiles. As AQPs can also be deleted in some eukaryotes, for example immortalized mammalian cells and trypanosomatids (Jeacock *et al*., 2017; Calvanese *et al*., 2018), it is clear that AQPs are non-essential, at least under some circumstances. Control of osmolarity therefore likely utilizes additional mechanisms in both pro- and eukaryotes. Below we will consider initially the evolution and origins of AQP paralogues in protists and then the uncovering of drug-related functions in trypanosomes.

## Evolution, functions and roles in protists

The evolution of the AQP family is surprisingly complex and at least three subfamilies with apparently distinct functions are recognized. These include AQPs able to translocate glycerol, others that only uptake water and a final third group, the superAQPs, that arose late in evolution. This latter subfamily is frequently intracellular, indicating a distinct function from the other members of the AQP family, which are usually located at the surface in most cells (Ishibashi *et al*., 2011), and are only found in metazoa. Significantly, the two ancestral forms are clearly differentiated in all prokaryotes, indicating an origin dating back to an early period of cellular life (Tong *et al*., 2019). The number of AQP paralogues in different species is highly variable, with land plants and vertebrates having the largest repertoires, as is the case for many other protein families.

There has been a considerable degree of expansion and contraction within specific lineages, or ‘churning’, with the result that functional differentiation between paralogues is difficult to predict (Ishibashi *et al*., 2020). Interestingly, in mussels (molluscs) there is evidence that expansion of AQP paralogues correlates with freshwater colonization events and hence facilitating adaptation to decreased environmental salinity (Calcino *et al*., 2019). Similar events may have facilitated tetrapod colonization of land habitats where desiccation is a considerable challenge (Finn *et al*., 2014) and underscores the importance of AQP evolution to life history.

**Fig. 2.**
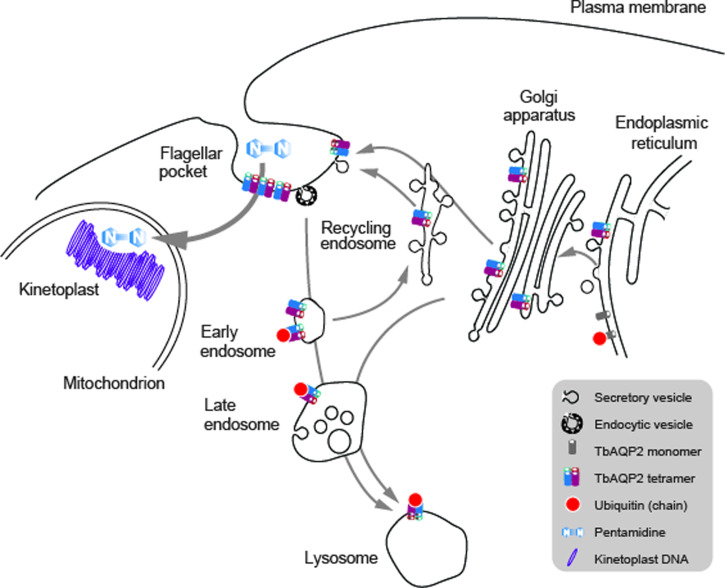
TbAQP2 trafficking, assembly and pentamidine uptake. Schematic of the trypanosome endomembrane system, focused on the region between the nucleus and flagellar pocket and encompassing the mitochondrion. AQP proteins are represented as open coloured cylinders, with the opening indicting the cell external/intracellular luminal face of the molecule. Note that both ER and endosomal molecules can become ubiquitylated (red dot). It is most likely that pentamidine enters the cell at the cell surface (see text) and is then translocated into the mitochondrion to interact with the kinetoplast (mitochondrial genome, purple circles) but he possibility that there is a contribution from endocytosis of AQP; pentamidine complexes remains a possibility.

In unicellular eukaryotes the number of AQP paralogues is comparatively small when compared with multicellular organisms and it has been proposed that the numbers of AQP paralogues are correlated somewhat with environmental complexity (von Bülow and Beitz, 2015). Most protist AQPs appear to be the more permissive glycerol-translocating forms that facilitate the uptake of solutes and waste compounds in addition to water. The free-living amoeba *Amoeba proteus* expresses a single AQP that is associated with the contractile vacuole (Nishihara *et al*., 2012), but by contrast there are four AQP paralogues in the social amoeba *Dictyostelium discoideum*, two of which are constitutively expressed and the remainder stage specific. Although there is evidence for roles in differentiation, none of the *D. discoideum* AQPs are exclusively water permeable and hence functions are not completely clear (Von Bülow *et al*., 2012). In the parasites *Plasmodium falciparum* and *Toxoplasma gondii*, each have a single AQP (Fig. 2) and this minimal repertoire may reflect intracellular life cycles and a more constant environment, *albeit* with considerable levels of complexity and differentiation events during life cycle progression, particularly for *P. falciparum.*

Amongst the kinetoplastids, *Leishmania major* has five AQPs, although only AQP1 has been studied in any detail. LmAQP1 is a wide permeability form localized on the flagellum and regulated by MAP kinase (Figarella *et al*., 2007; Mandal *et al*., 2012; Sharma *et al*., 2015). The remaining *L. major* AQPs are less well uncharacterized, but at the sequence level more closely resemble the plant tonoplast intrinsic protein (TIP) AQP subclass. Four of the five AQP genes in *L. donovani* retain canonical gating motifs, but in one paralogue this is mutated to NPM-NPA. All four of the conventional AQPs are suggested as intracellular as is the case for the TIP AQPs of plants, but significantly LdAQP1 is likely to permit translocation of large solutes (Biyani *et al*., 2011). Antimonial-containing drugs remain a first line treatment against *Leishmania* in many parts of the world (Field *et al*., 2017) and in laboratory derived strains of *Leishmania mexicana* AQP1 can restore antimonial uptake to resistant cells (Marquis *et al*., 2005). No obvious genome level changes to gene copy number or sequence accompany resistance but is potentially a post-transcriptional modulation of AQP1 mRNA level. Changes to expression of AQP1 have been demonstrated in multiple species where resistance was derived in the laboratory (Lin *et al*., 2008; Barrera *et al*., 2017). However, it is also clear that there is a less compelling case for association of altered AQP1 expression and drug resistance in clinical isolates.

The American trypanosome, *Trypanosoma cruzi* also has four TIP-like AQPs, representing the entire repertoire in that organism and these are associated with the contractile vacuole and acidocalcisomes (Montalvetti *et al*., 2004). *Trypanosoma brucei* has three AQPs; AQP1 is shared with other kinetoplastida, while AQP2 and AQP3 arose from a recent gene duplication in the African trypanosome lineage and remain contiguous.

In addition to interactions between *trans*-membrane domains, two major selectivity filters restrict the molecular weights and properties of the solutes being translocated by AQPs and that can effectively pass through the central pore; these are the ar/R and NPA/NPA motifs (Fig. 1) (Beitz, 2005; Baker *et al*., 2013; Verkman *et al*., 2014; Munday *et al*., 2015; Fairlamb and Horn, 2018). *Trypanosoma brucei* AQP1 and AQP3 display the internal arrangements in the protein pore observed in canonical AQPs, including the canonical ‘NPA’ within two half *α*-helices and a narrower ‘aromatic/arginine’ (ar/R) motif (Beitz, 2005). Interestingly, TbAQP2 does not retain this canonical configuration, displaying an unconventional ‘NPS/NSA’ filter motif and rearrangement in the ar/R motif that is replaced by a neutral leucine at position 264 (L264), followed by aliphatic, rather than aromatic, residues (A88, I110, V249 and L258), which are equivalent to the ‘IVLL’ motif observed in the selectivity pore of canonical AQPs (de Groot and Grubmuller, 2001; Baker *et al*., 2013; Quintana *et al*., 2020). These structural features indicate that TbAQP2 can accommodate larger solutes through the selectivity pore (Uzcategui *et al*., 2004).

These examples demonstrated that AQP evolution is highly plastic, with the creation of additional paralogues, facilitating altered specificity. Hence, the AQP family contributes to surviving environmental complexity and exploitation of new ecological niches, with a considerable impact on the life history of the earth. However, the absence of AQPs from many lineages or a genetic demonstration of essentially in many organisms serves to underscore the challenges remaining for the full understanding of AQP function.

## TbAQP2 and multidrug resistance

The treatment of sleeping sickness relies on drugs to clear first- or second-stage infections, and the choice of drug depends on the capacity to penetrate the blood–brain barrier (BBB) (Denise and Barrett, 2001; Steverding, 2010; Fairlamb and Horn, 2018). Of these, pentamidine and melarsoprol represent two of the most potent drugs currently used to treat first- and second-stage diseases, respectively, displaying low nanomolar 50%-effective growth-inhibitory concentration (EC_50_) (Denise and Barrett, 2001; Bray *et al*., 2003; Barrett *et al*., 2007; Fairlamb and Horn, 2018). Pentamidine, an aromatic diamidine, is used to treat first-stage (haemolymphatic stage) *T. gambiense* HAT (Denise and Barrett, 2001; Barrett *et al*., 2007; Baker *et al*., 2013). This compound binds nucleic acids with high affinity, leading to accumulation by, and ultimately destruction of, the kinetoplast (Mathis *et al*., 2006; Baker *et al*., 2013; Gould and Schnaufer, 2014; Al-Horani *et al*., 2019; Kennedy and Rodgers, 2019). However, pentamidine is unable to reach the central nervous system (CNS), in part due to its high affinity interactions with serum proteins, charge and relatively high retention in tissues and is therefore ineffective for the treatment of second-stage meningoencephalic HAT (Barrett *et al*., 2007; Maclean *et al*., 2012). Melarsoprol, on the contrary, is an arsenical compound used for the treatment of second-stage HAT, including *T. rhodesiense* HAT (Fairlamb *et al*., 1989; Keiser *et al*., 2000; Field *et al*., 2017). This compound is thought to be metabolized to melarsen oxide prior to uptake by African trypanosomes, leading to the formation of a stable adduct with trypanothione known as Mel T (Burri *et al*., 1993, 1994; Fairlamb and Horn, 2018). Melarsoprol penetrates the BBB comparatively more effectively than pentamidine, reaching the minimum concentration required for parasite clearance in the CNS (Mäser *et al*., 1999; Stewart *et al*., 2010). Melasoprol also displays reactive encephalopathy in ~10% of patients, which is frequently fatal (Fairlamb and Horn, 2018).

Given the limited repertoire of drugs available for treatment it is perhaps not surprising that resistance to these compounds has been frequently observed in endemic countries. Indeed, diamidine-arsenical cross-resistance was initially reported in the 1940s, suggesting that mechanisms of uptake and/or action were common to these otherwise divergent chemical compounds, but with the molecular details poorly understood. The identification of the pentamidine/melarsoprol transporter has been a serendipitous process. Initial studies in cross-resistance in laboratory strains (Bernhard *et al*., 2007; Bridges *et al*., 2007; Graf *et al*., 2015*a*) and field isolates (Shahi *et al*., 2002; Alsford *et al*., 2012) from relapsed patients identified the gene encoding for the purine transporter responsible for drug uptake as *T. brucei* adenosine transporter 1 (TbAT1). In addition to TbAT1, the high-affinity pentamidine transporter (HAPT1) (Bernhard *et al*., 2007) as well as the ATP-binding cassette transporter MRPA (Baker *et al*., 2012) were also proposed to mediate drug resistance by various mechanisms, but neither explained the drug resistance levels observed in field isolates (Baker *et al*., 2013).

Using genome-wide RNAi-mediated genetic screening and functional assays, the locus encoding the closely related AQP2 and AQP3 was identified as a *bona fide* hit for pentamidine/melarsoprol cross-resistance (Graf *et al*., 2015*b*). Further biochemical and genetic manipulation studies demonstrated that deletion of AQP2, but not AQP3, led to a significant increase in the EC_50_ of both compounds, mirroring the behaviour observed in previously generated laboratory strains and field isolates (Munday *et al*., 2014; Graf *et al*., 2015*b*; Song *et al*., 2016). Other observations such as localization to the flagellar pocket in the bloodstream form (Munday *et al*., 2014; Graf *et al*., 2015*b*; Song *et al*., 2016; Quintana *et al*., 2020), as well as the unusual pore structure discussed above, led to the hypothesis that pentamidine and melarsoprol are likely to interact with high affinity to AQP2 located in the flagellar pocket (Alghamdi *et al*., 2020), posing the question of how these compounds are internalized and also the mechanisms for resistance.

## Endocytosis or membrane uptake: competing models for drug entry

Suggesting that the role of a channel protein is not the primary mechanism for pentamidine to access the trypanosome cytoplasm may seem to be a straw man, but this possibility has been proposed. Specifically, as AQP2 binds pentamidine with high affinity at the first selectivity pore, the possibility that AQP2 is a receptor for uptake by endocytosis is not unreasonable (Fig. 2) and could act as a parallel to ISG75-mediated uptake of suramin (Graf *et al*., 2015*b*). This model was further supported by reports demonstrating that pentamidine binds AQP2 with nanomolar affinity, thus potentially acting as a highly selective inhibitor of AQP2 (Fig. 2) (Alghamdi *et al*., 2020). However, consideration of structural features of the pore do support TbAQP2 acting as a channel for larger and more structurally flexible solutes including pentamidine (Petersen and Beitz, 2020). In the endocytosis model, ubiquitination of TbAQP2 at the flagellar pocket is central for subsequent ubiquitination-mediated intracellular trafficking and delivery to intracellular organelles such as the lysosome. Indeed, TbAQP2 forms a stable homomultimeric complex in the flagellar pocket where ubiquitination is likely to take place on individual monomers (Quintana *et al*., 2020).

The opposing membrane uptake model proposes that pentamidine, and potentially melarsoprol, are taken up *via* the intrinsic channel properties of TbAQP2. Indeed, a recent report demonstrates that drug permeation is possible due to a highly conserved amino acid motif in the central pore architecture of TbAQP2, facilitating the passage of ‘high’ molecular weight solutes (Alghamdi *et al*., 2020). This was demonstrated by TbAQP3 mutants containing the amino acids of the selectivity pore from TbAQP2 possessing increased capacity for pentamidine uptake (Alghamdi *et al*., 2020). Moreover, pentamidine permeation through TbAQP2 seems to be further aided by the intrinsic membrane potential and is not abrogated by partially blocking endocytic uptake (Alghamdi *et al*., 2020; Quintana *et al*., 2020), *albeit* at a rate that is considerably slower than for lower molecular weight solutes, which in essence implies a leak in the AQP2 permeability barrier.

Concerning the likely site for pentamidine uptake, there is no evidence that endocytosis or post-translational modification of AQP2 is required. Specifically, additional genes identified from the genome-wide RNAi screen identified a kinase and phosphatase for melarsoprol and pentamidine respectively, as well as one unique hypothetical each (Alsford *et al*., 2012). None of these genes have evidence for roles in ubiquitylation, endocytosis or trafficking in general, suggesting that translocation of drugs from the surface is sufficient for toxicity and that blocking ubiquitylation or endocytosis does not offer resistance. However, it needs to be acknowledged that a role for endocytosis that is overshadowed by the channel-mediated mechanism, remains a possibility.

## Stability and folding of TbAQP2 contribute to pentamidine resistance

In common with most membrane proteins, AQPs undergoing translation are inserted into the endoplasmic reticulum through the Sec61 translocon and assisted in folding *via* a cohort of chaperones (Pitonzo and Skach, 2006). Given that most AQPs are also glycoproteins it is likely that the calnexin/calreticulin quality control system is involved in monitoring quality and rapidity of folding. Importantly, formation of homotetrameric complexes is important for AQP stability and the formation of heterotetrameric complexes has not been observed (Duchesne *et al*., 2002; Furman *et al*., 2003). The residues responsible for this specificity are not clear, but AQP tetramers can assemble into higher order quasi-crystalline arrays (Kitchen *et al*., 2016). Furthermore, there are clear differences in the stabilities of the water and solute permeable AQP tetramers with the former exhibiting greater stability than the latter and likely due to features within the final two *trans-*membrane domains and loops D and E (Lagrée *et al*., 1998; Duchesne *et al*., 2002; Buck *et al*., 2007; Kitchen *et al*., 2016), *albeit* with the functional consequences, if any, unclear. Significantly the folding pathway is not identical for all AQPs, being controlled at least partly by sequences within the second *trans*-membrane domain (Carrington *et al*., 2010). Finally, mammalian AQPs are both phosphorylated and ubiquitylated, with at least the latter contributing to protein turnover, endocytosis and quality control (Kamsteeg *et al*., 2006; Mandal *et al*., 2012; Sharma *et al*., 2015; Quintana *et al*., 2020). Although it is most likely that similar pathways operate in trypanosomes, with direct evidence for ubiquitylation and most of the relevant folding chaperones present, the precise mechanisms of AQP maturation, at least in African trypanosomes, remain to be investigated in detail (Field *et al*., 2010; Tiengwe *et al*., 2016*a*, 2016*b*).

To understand folding, stability and trafficking of AQP2 in *T. brucei* we examined sequence-dependence and *trans*-membrane domain exchange designed to mimic natural AQP2/3 chimeras expressed in a triple null background (Jeacock *et al*., 2017; Quintana *et al*., 2020). TbAQP2 forms both tetramers and tetramers of tetramers and is degraded in the lysosome by a ubiquitin-dependent process (Fig. 2) (Quintana *et al*., 2020). Attempts to influence ubiquitination by mutating cytoplasmic lysine residues unexpectedly reduce stability rather than preventing lysosomal targeting (Quintana *et al*., 2020). This is due to reduced folding and tetramerization efficiency, which triggers ER-associated degradation, indicating a failure to complete quality control (Quintana *et al*., 2020). Perhaps the most significant finding is that chimerical TbAQP2/3 proteins also lead to impaired folding and reduced stability (Quintana *et al*., 2020). This was also the case for constructs mimicking chimeras found in trypanosomes from patients where pentamidine treatment had failed.

Clearly rigorous quality control mechanisms operate within the ER of *T. brucei*, but with a consequence that mutations in the non-essential AQPs can render parasites refractory to treatment. Moreover, the instability of AQP2 is likely an underlying cause of pentamidine treatment failure while the production of chimeric forms is potentially a high frequency event and stems directly from generation of contiguous paralogues initially derived by gene duplication; presumably the chimeras have poor folding capability due to mismatch between the N- and C-terminal regions.

## Concluding remarks

**Fig. 3.**
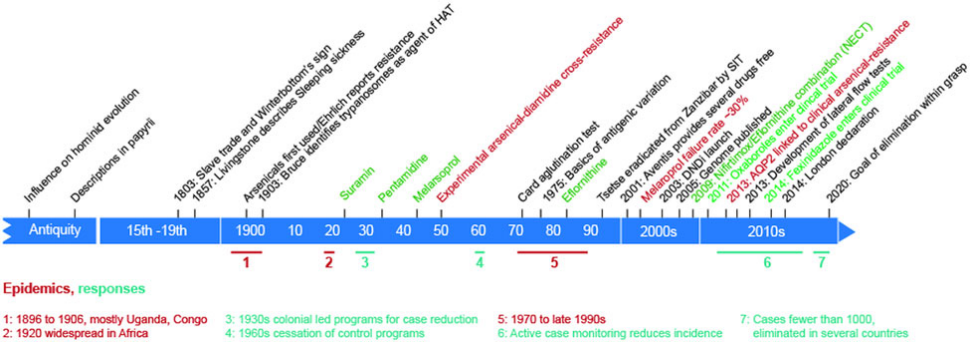
Major events in the history of African trypanosomiasis. Annotations above the timeline in black indicate major cultural, historical and management events with a bearing on trypanosomiasis. The influence on hominid evolution is inferred from impact on savannah ecosystems. Annotations in green indicate introduction of chemotherapeutic agents and in red emergence of resistance mechanisms and related advances in molecular understanding. Annotated beneath the timeline are periods of major change in the incidence of trypanosomiasis, in red for periods of epidemics and teal for control measures.

Remarkable advances to understanding mechanisms for classical therapies against African trypanosomes, as well as development of new drugs and the successes of public health programmes, auger well for the control of both human and animal African trypanosomiasis. Remarkably, we now have considerable understanding of pentamidine and melarsoprol uptake as well as mechanisms for resistance. The evolutionary history of trypanosome AQPs reveals both how pentamidine sensitivity arose, with a specifically broad-spectrum AQP2, and resistance arising from recombination. Placed in context (Fig. 3) the millennia-old relationship between trypanosomes and humans has been complex, with periods where one organism had the upper hand and then the other. Recently, humans have been in the ascendant, with case numbers having dropped precipitously and even exceeding the WHO roadmap predictions. Indeed, several countries previously considered endemic have reported no cases for several years. It can only be hoped that the advances made in the last decade are not eroded by the COVID-19 pandemic, which threatens to undermine global progress on many fronts, including the control of infectious diseases (http://hdrundp.org/en/2020-report).
